# Therapeutic Application of mRNA for Genetic Diseases

**DOI:** 10.1002/wnan.70019

**Published:** 2025-05-26

**Authors:** Paul J. L. Schürmann, Stijn P. E. van Breda Vriesman, Jose A. Castro‐Alpízar, Sander A. A. Kooijmans, Edward E. S. Nieuwenhuis, Raymond M. Schiffelers, Sabine A. Fuchs

**Affiliations:** ^1^ Division of Metabolic Diseases Wilhelmina Children's Hospital, University Medical Center Utrecht Utrecht the Netherlands; ^2^ Regenerative Medicine Center Utrecht Utrecht the Netherlands; ^3^ Erasmus MC Rare Disease Center, Department of Pediatrics, Erasmus MC University Medical Center Rotterdam Rotterdam the Netherlands; ^4^ CDL Research, University Medical Center Utrecht Utrecht the Netherlands; ^5^ Nanocell Therapeutics Utrecht the Netherlands

## Abstract

While gene therapy has been at the center of an active research field for decades, messenger RNA (mRNA) has long been considered unsuited for therapeutic application due to challenges with stability, immunogenicity, and delivery. Where gene therapy focuses on providing the desired genetic code, mRNA can directly provide the instructions encoded in the corresponding gene. This review aims to explore recent advances in mRNA therapies, building on the success of mRNA COVID‐19 vaccines, and extend these insights to the potential treatment of rare genetic diseases. We follow the “outside‐in” trajectory of mRNA therapies from administration to intracellular function, focusing on carrier systems such as lipid nanoparticles and virus‐like particles, mRNA modifications, and the potential and challenges for clinical applications. To treat rare diseases, different approaches can be envisioned, including chronic or acute delivery of mRNAs encoding functional enzymes for enzyme deficiencies and delivery of CRISPR/Cas9–based gene‐editing tools for gene correction. These different approaches determine safety and immunological considerations. By exploring genetic, technical, and therapeutic aspects, this review highlights the potential and current challenges of mRNA therapies to address the large unmet needs in rare genetic disorders.

## Graphical abstract

Overcoming challenges in mRNA therapeutics for rare genetic diseases, such as protection against degradation and immune clearance, organ targeting, and reducing intracellular immunotoxicity with delivery vehicles and mRNA modifications for effective expression and lifespan.

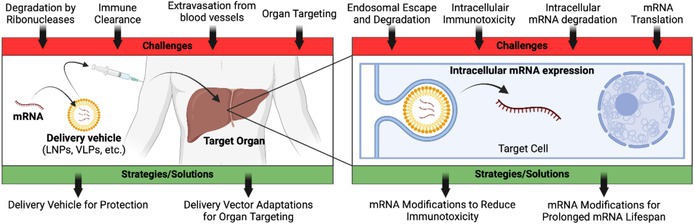

## 1 Introduction

As the main cellular effector molecules, proteins are attractive therapeutic targets. They can be introduced into the body directly as proteins but also indirectly by providing the encoding genetic information. The use of genes and proteins for therapeutic applications has been a central research topic for decades. By now, proteins are well‐established therapeutic agents, such as insulin for diabetes, enzymes for metabolic diseases, and antibodies for blocking or engaging specific receptors. Similarly, gene therapies are gradually evolving into clinical therapies. The step between genes and proteins involves messenger ribonucleic acid (mRNA). Unlike DNA‐based treatments, which pose risks of genomic integration, mRNA offers a safe, efficient, and relatively simple alternative for therapeutic protein production. However, the therapeutic use of mRNA has long remained impossible due to instability, immunogenicity, and delivery challenges.

This status quo changed during the coronavirus 2019 (COVID‐19) pandemic with the unprecedented rapid development of the first two COVID‐19 mRNA vaccines (BioNTech BNT162b2 and Moderna mRNA‐1273) (Baden et al. 2021; Polack et al. 2020). These lipid nanoparticle (LNP)–based mRNA vaccines achieved protective antibody titers in 95% and 94.1% of recipients, respectively, and promptly became the most widely used mRNA therapeutics (Baden et al. 2021; Polack et al. 2020). This was not an overnight achievement. To overcome the instability, immunogenicity, and high costs of mRNA therapeutics, innovative engineering technologies were required for mRNA sequence optimization, chemical modifications, and purification. In addition, sophisticated delivery vehicles were required to protect the mRNA from undesired recognition by the immune system and digestion by endogenous enzymes and to allow delivery and translation of the mRNA in the cells of interest (X. Huang et al. 2022). The pioneering work on mRNA modifications by Katalin Kariko and Drew Weissmann laid the foundation for this first therapeutic use of mRNA and resulted in the 2023 Nobel Prize in Physiology or Medicine. The strength of mRNA lies in the provision of cells with the relatively simple blueprint, consisting of only four different nucleotides, to produce specific proteins using the body's own machinery. This establishes mRNA as a platform technology, where the mRNA sequence can be easily modified without changing its physicochemical characteristics, thereby allowing rapid adaptations, as evidenced by the rapid generation of new vaccines to protect against evolving COVID strains (Zhou et al. 2023). In addition to therapeutic use in vaccine development, mRNA is promising for cancer treatment, for enzyme replacement, and for delivery of gene editors for monogenic diseases. These different applications involve different requirements, for example, short expression of a viral or tumor protein to induce an immune reaction, lifelong expression to restore a protein deficiency, or a one‐time temporary expression of a gene editor to generate permanent gene correction. While the duration of expression and the effects of immune responses present varying challenges and opportunities for these different applications, “getting there,” meaning organ‐ and cell‐specific targeting, still remains a ubiquitous challenge for clinical application.

We here focus on monogenic diseases. These originate from DNA mutations that result in the production of incorrect or deficient mRNA and in a deficiency of the corresponding protein. Depending on the gene, the mutation, the protein and the cell or organ in which this occurs, essentially any symptom can arise in any organ at any age. Therapeutic options are generally limited. Each of the 7000 monogenic diseases are rare (affecting less than 6.5 in 10.000 people according to the World Health Organization, Aronson 2006). However, when combined, they affect around 3% of the world population (Hieter and Boycott 2014). Of these, 70% start in childhood and result in severely reduced quality of life and life expectation (Hemilä 2000). The discovery and development of effective treatments for rare genetic diseases has seen limited progress, when compared to treatments for more common conditions. At present, around 50 new therapies for rare genetic diseases are approved around the world each year. “At this rate, it would take more than 100 years to develop a single treatment for every rare disease estimated to exist worldwide” (Willmer 2022). This underscores the desire for innovative therapeutic approaches tailored to the unique technical and practical challenges posed by rare diseases. In this respect, gene‐ and mRNA‐based therapies may provide a promising therapeutic option for these monogenic diseases. However, functional delivery of mRNA into the affected target cells is crucial and “getting there” remains a major roadblock for therapeutic application of mRNA in monogenic diseases.

In this review, we offer an overview of the state‐of‐the‐art mRNA production and delivery techniques with an “outside‐in” perspective, following the pathway of the mRNA therapy from administration in the extracellular environment to executing its function intracellularly. First, we address the challenges of mRNA stability in the extracellular environment, highlighting the need for encapsulation in nanocarrier‐based delivery systems, which can be targeted to specific cell types (Section 2). The mRNA then needs to be taken up by these cells, escape degradation and be translated intracellularly (Section 3). We will conclude with a discussion of the therapeutic potential of mRNA for enzyme replacement and gene‐correction therapies for rare diseases (Section 4).

## 2 Delivery of mRNA

### 2.1 The Necessity of mRNA Protection and a Delivery System

The concept of nucleic acid‐based therapies is based on the observation that direct injection of pure RNA and DNA into mouse skeletal muscle resulted in the expression of the encoded protein (Wolff et al. 1990). However, for systemic application of mRNA, mRNA proved too unstable and immunogenic (Karikó et al. 2008; Sahin et al. 2014; Wolff et al. 1990; Zarghampoor et al. 2019). The first challenges for therapeutic mRNA occur right after administration in the extracellular environment in the human body, when mRNA is exposed to ribonucleases. These enzymes are omnipresent and a single cut may inactivate mRNA. Simultaneously, mRNAs are threatened by uptake and degradation by immune cells (Evers et al. 2018). Even when mRNAs are able to circumvent these challenges, the large size and negative charge of mRNA molecules complicate passage through anionic membranes, hindering internalization into target cells (Hajj and Whitehead 2017).

Further development of mRNA therapies was nevertheless pursued because of several advantages associated with mRNA therapies, including the simple structure of RNA, direct translation in the cytosol, transient expression, and limited risks of integration into the DNA (Karikó et al. 2008; Oh and Kessler 2018; Sahin et al. 2014; To and Cho 2021; Zarghampoor et al. 2019). For vaccine development, direct injection of naked mRNA in specific buffers was explored. Although mRNA cannot naturally diffuse through cell membranes, it can be internalized by cells through mechanisms such as macropinocytosis (Selmi et al. 2016) or mechanical forces like hydrostatic pressure (Stewart et al. 2018). This may be a feasible approach for local administration, such as intranodal or intradermal injection in the highly immunologically active environment of the skin and lymph nodes (Pardi et al. 2018). Complexing the negatively charged mRNA with peptides with positively charged amino acids like lysine and arginine may provide an additional strategy to protect mRNA from degradation by RNAses and induce adjuvant activity for vaccines (Pardi et al. 2018).

However, for mRNA therapeutics for the majority of other applications, efficient and safe delivery vectors are essential to mitigate extracellular biodegradation and facilitate effective cellular uptake in organs affected by rare diseases. Currently, LNPs and virus‐like particles (VLPs) are the most advanced vehicles for clinical mRNA delivery.

### 2.2 Lipid‐Based Particles for Therapeutic mRNA Delivery

Lipid‐based particles, in particular LNPs, have evolved as efficient mRNA delivery vectors. Their earliest predecessors are liposomes, which are now considered the first nanomedicine delivery platform (R. Tenchov et al. 2021). Liposomes are nanosized vesicles composed of a phospholipid bilayer surrounding an aqueous core. They can transport a wide range of pharmaceutical molecules, ranging from hydrophobic to hydrophilic molecules including proteins, nucleic acids, and conventional small molecular weight drugs (R. Tenchov et al. 2021). The therapeutic potential of liposomes was recognized rapidly after their discovery. They were primarily utilized for transporting small molecules. The approval of doxorubicin‐loaded liposomes to treat sarcomas in the late 1990s marked a significant milestone in nanomedicine (Gabizon et al. 1989). However, further progress in developing liposomal treatments remained challenging, especially for biotechnological drugs like mRNA and ribonucleoproteins (RNPs). Encapsulation and delivery of negatively charged nucleic acids with conventional liposome structures were not effective (Fan et al. 2021). Therefore, cationic (CL) and ionizable lipids (IL) were incorporated to electrostatically complex and protect mRNA, drastically improving encapsulation efficiency. In addition, improvements in production methods allowed modulation of particle structure. Resulting lipid particles with an electron‐dense core instead of the traditional aqueous core are now referred to as LNPs. The state‐of‐the‐art method for preparation of LNPs consists of rapid mixing of lipids in ethanol with aqueous mRNA solutions. These microfluidic mixing techniques result in homogeneous particle sizes with excellent reproducibility (Evers et al. 2018).

### 2.3 Physicochemical Properties of LNPs to Improve Stability

**FIGURE 1:**
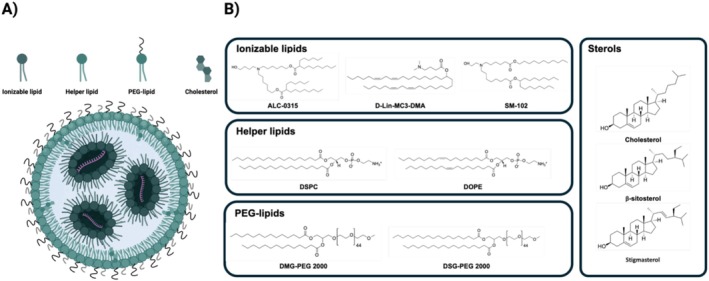
Lipid nanoparticle composition: (A) lipid nanoparticle composition consisting of ionizable lipids, helper lipids, cholesterol and PEGylated lipids; (B) common lipid structures in approved lipid nanoparticles.

After systemic administration, LNPs offer greater mRNA stability and the potential for long circulation compared to naked mRNA. Important properties in this process involve the lipid composition, surface properties, and size distribution (Evers et al. 2018). Currently, the most effective LNP formulations typically incorporate four types of lipids: ILs, helper lipids, polyethylene glycol (PEG) lipids, and cholesterol (Kauffman et al. 2015) (Figure 1A) and the function of the individual types of lipids will be discussed in the following paragraphs. The resulting lipid composition determines the particle size, morphology, surface properties, and encapsulation efficiency (Blanco et al. 2015; Kanasty et al. 2013). Multiple formulations are currently used and depend on the specific application, with most formulations naturally accumulating in the liver (Evers et al. 2018).

#### 2.3.1 Cationic/Ionizable Lipids

Incorporation of positively charged lipids supports the formation of stable lipid‐nucleic acid complexes within the LNP and leads to efficient encapsulation of mRNA (Miwa et al. 2024). Depending on the type of headgroups, these lipids are either categorized as cationic or ionizable. CLs have permanently positively charged hydrophilic headgroups. This cationic charge facilitates electrostatic interaction with the negatively charged backbone of nucleic acids, aiding in the encapsulation of nucleic acid cargo within the electron‐dense LNP core. However, the permanent cationic charge of these lipids may also lead to undesired toxicity (S. Cui et al. 2018), for example, through aggregation with negatively charged red blood cells (L. Cui et al. 2022). ILs are also hydrophilic but are less toxic due to their pH‐dependent cationic character (Eygeris et al. 2022). At low pH, ILs are protonated, resulting in a positive charge, but at physiological pH, they are neutral, which helps to prevent adsorption of negatively charged biomolecules in the physiological environment, thereby preventing immunological clearance and unwanted toxicity. Prevention of adsorption of plasma components also contributes to more effective binding of LNPs to their target cells (Sebastiani et al. 2021). After internalization in the target cells, LNPs are trapped in the endosomal lumen in which the pH is lower than in the extracellular environment. In this low pH, the ILs are (re‐)protonated and become positively charged and may disrupt or destabilize the endosomal membrane by formation of transient pores and facilitate endosomal escape and nucleic acid release (Hou et al. 2021).

While the headgroups are most important for encapsulation efficiency and biocompatibility, the linkers and the hydrophobic tails also define LNP characteristics. The linker, which connects the headgroup with the hydrophobic tail, influences biodegradability, successful delivery of the cargo, and cytotoxicity of the LNPs (Eygeris et al. 2022). Linkers can be biodegradable or nonbiodegradable. Biodegradable linkers typically incorporate functional groups that are susceptible to enzymatic or chemical cleavage under physiological conditions (Buschmann et al. 2021; Maier et al. 2013). The presence of ester groups, for example, increases the biodegradability of LNPs. Ester linkers are stable at physiological pH, yet they are susceptible to enzymatic hydrolysis by esterases or lipases in tissues and intracellular compartments (Maier et al. 2013). Biodegradable linkers are generally preferred due to rapid clearance in vivo, thereby allowing for multiple dosing and minimizing risks of side effects (Eygeris et al. 2022). Currently approved LNP therapeutics, ALC‐0315 and SM‐102, use ILs (Figure 1B) with ester linkers (Eygeris et al. 2022; Jörgensen et al. 2023). Another clinically approved ionizable lipid, DLin‐MC3‐DMA, is not biodegradable despite ester linkage (Jörgensen et al. 2023). Nonbiodegradable linkers typically consist of more stable chemical bonds that are less susceptible to enzymatic cleavage or hydrolysis in biological environments. These linkers may include structures such as ethers, aromatic rings, or amides (L. Yang et al. 2022), and result in greater stability which may be of interest for specific functionalities such as controlled release, targeting of specific organs, or increased LNP stability.

The hydrophobic tails are important for their role in LNP assembly and potency through regulation of lipophilicity, fusogenicity, pKa, and fluidity. The tails can differ in number, length, sidechains, and saturation, thereby affecting LNP fluidity, stability, cellular uptake, and mRNA delivery (Heyes et al. 2005; Sabnis et al. 2018). As an example, the difference in tail sidechains (Figure 1B) of the ionizable lipids SM‐102 and ALC‐0315 in the COVID‐19 vaccines Spikevax (Moderna) and Comirnaty (Pfizer), respectively, may contribute to the higher pKa of SM‐102, and consequently more efficient endosomal escape (L. Zhang et al. 2023).

Use of CLs/ILs in LNPs has revolutionized mRNA therapeutics by overcoming limitations in encapsulation efficiency and stability. ILs are generally better tolerated and safer than the more cytotoxic CLs with their constant positive charge (Sun and Lu 2023). However, ILs may impose immunogenic risks, as stimulation of Toll‐like receptors (TLRs) and increased concentrations of pro‐inflammatory cytokines were observed with the use of DLin‐MC3‐DMA in clinically approved siRNA‐LNPs (Bitounis et al. 2024).

#### 2.3.2 Helper Lipids

The efficiency and stability of LNPs in delivering nucleic acids hinge significantly on the presence of helper or structural phospholipids (Figure 1B). Comprising headgroups and tails, these phospholipids can be tailored to the required function within the LNP. The two most commonly used helper lipids are 1,2‐distearoyl‐sn‐3‐phosphocholine (DSPC) and 1,2‐dioleoyl‐sn‐glycero‐3‐phosphoethanolamine (DOPE). DSPC is characterized by saturated tails and a large headgroup, promoting a cylindrical geometry. This lipid is the cornerstone in clinically approved LNPs, for example, those employed in mRNA COVID‐19 vaccines (R. Zhang et al. 2021). In contrast, DOPE's smaller headgroup and unsaturated tails induce a conical shape, conferring improved fusogenic properties (Evers et al. 2018; Seo et al. 2023). Due to their diverging properties, helper lipids are not universally compatible with ionizable lipids, which can have major effects on LNP transfection capacity and stability (Kulkarni et al. 2017, 2019).

#### 2.3.3 Cholesterol

Cholesterol (Figure 1B) is required for the stability of LNPs and efficiency of RNA delivery. As an essential component of eukaryotic membranes, cholesterol stabilizes internal and external lipid structures within LNPs (J. Zhang et al. 2019). Incorporation of increasing cholesterol concentrations in LNPs leads to tighter packing, reduced membrane permeability, and decreased fluidity. The structural rigidity provided by cholesterol is vital for maintaining LNP integrity until target cells are reached and enabling effective endosomal escape following membrane fusion (Sakurai et al. 2001; Semple et al. 1996; B. G. Tenchov et al. 2006). Accordingly, reduced cholesterol incorporation was found to correlate with decreased mRNA expression in target organs in mice (Kawaguchi et al. 2023). By modifying the structure of cholesterol, delivery efficiency can further be modulated, as demonstrated in LNPs where β‐sitosterol (Figure 1B), a cholesterol analog, resulted in higher intracellular uptake and increased endosomal escape compared to LNPs containing cholesterol (Patel et al. 2020). Stigmasterol, another analog with unsaturated tails (Figure 1B), resulted in lower mRNA encapsulation (Patel et al. 2020). Use of cholesterol and analogs thus influences LNP characteristics, but the effects of cholesterol modifications are still relatively poorly understood. Availability, production costs, and batch‐to‐batch variability of the cholesterol derivatives may contribute to the choice of the cholesterol analog (Eygeris et al. 2022).

#### 2.3.4 PEG‐Anchored Lipids

PEG lipids are integral to the composition and function of LNPs and enhance colloidal stability and prevent opsonization of plasma proteins. They localize at the outer shell of LNPs due to their hydrophilicity (Ongun et al. 2024). Incorporation of PEG lipids allows precise control over the size and zeta potential of LNPs (Jung et al. 2022). They contribute to particle stability and have a profound influence on the pharmacokinetics of the LNPs, affecting half‐life in the circulation by preventing the adsorption of opsonins that enable immune recognition and clearance (Bao et al. 2013). Furthermore, PEG lipids can be functionalized by bioconjugation with targeting ligands or biomacromolecules for drug delivery to specific cells or organs. Characteristics such as molar mass and lipid length of the PEG lipids determine LNP behavior. Most of the clinically applied LNPs use PEG2000 conjugated to a lipid with two saturated C14‐acyl chains, such as dimyristoyl glycerol (DMG) (Figure 1B). This C14‐acyl chain seems to provide the optimal compromise between inertness and reactivity. Longer acyl chains (e.g., C18‐disteaoryl glycerol) stabilize the particle for prolonged periods of time, increase circulation time, and oppose interactions with cells. However, C18 PEG lipids also hamper interaction with the endosomal membrane, potentially promoting degradation before endosomal escape. Shorter acyl chains rapidly dissociate upon administration, causing rapid opsonization and clearance. The C14 compromise causes a gradual dissociation that for most LNPs coincides with opsonization with apolipoprotein E, favoring uptake by hepatocytes (Figure 1B) (Berger et al. 2023; Hou et al. 2021). PEG lipids thus play an important role in LNP stability and pharmacokinetics. Changes in quantity and properties of PEG‐lipids can tremendously affect LNP delivery potency (Eygeris et al. 2022; Seo et al. 2023).

#### 2.3.5 LNP Immunogenicity

Despite significant advancements with clinically used LNPs, numerous studies highlight LNP‐induced inflammation as a major hurdle for further therapeutic applications (Ndeupen et al. 2021; Omo‐Lamai et al. 2024; Tahtinen et al. 2022; Walsh et al. 2020). Identifying the optimal LNP composition remains a challenge due to the limited understanding of the relationship between LNP constituents and immune responses. For instance, the clinically approved ionizable lipid SM‐102 is known to be immunostimulatory, whereas Dlin‐MC3‐DMA demonstrates relative inertness (Tahtinen et al. 2022). Yet, the use of Dlin‐MC3‐DMA‐LNPs (Onpattro) still requires pretreatment with corticosteroids and antihistamines to suppress infusion reactions, while this is not needed for SM‐102. The immunogenicity of ionizable lipids appears to be closely linked to their chemical structure. A study investigating the immunogenicity of LNPs with varied headgroups and tails revealed that headgroups, in particular, influence immunogenicity significantly (Chaudhary et al. 2024). LNPs containing amine headgroups were found to be immunogenic, activating both the innate and adaptive immune systems through enhanced binding to TLR4 and CD1d (Chaudhary et al. 2024; Sharma et al. 2024).

Despite their immunogenicity, amine headgroups remain indispensable for the ionizability of approved lipids, posing a significant challenge in their use. Strategies to mitigate this often focus on adjusting the physicochemical characteristics of LNPs—such as optimizing lipid ratios, adjusting mRNA size, adding adjuvants, or modifying the administration speed and duration. Additionally, as seen with Dlin‐MC3‐DMA, pretreatment with immunosuppressive agents can also help reduce immune reactions (Lee et al. 2023).

The immunogenicity of PEG‐lipids also raises concerns. Upon repeated dosing of LNPs or exposure to other PEGylated moieties, anti‐PEG antibodies can be formed. These antibodies accelerate clearance of new doses of LNPs (the so‐called “ABC‐effect”) and reduce the expression of encapsulated mRNA (Besin et al. 2019; Bevers et al. 2022). In addition, anti‐PEG antibodies on the surface of LNPs can form a scaffold for complement activation, potentially causing degradation of encapsulated cargo (Estapé Senti et al. 2022) and hypersensitivity reactions involving complement system activation have been reported for PEG lipids (Zong et al. 2023). To address these concerns, alternatives to PEG and biodegradable components are explored for LNP formulations. Polysarcosine is one of these alternatives, which may replace PEG in many applications without significant impact on the LNP properties (Hu et al. 2018). Another approach may involve a rationally designed dosing regimen that reduces the formation of anti‐PEG antibodies (Besin et al. 2019).

### 2.4 LNP Delivery to the Target Cells of Interest

**FIGURE 2:**
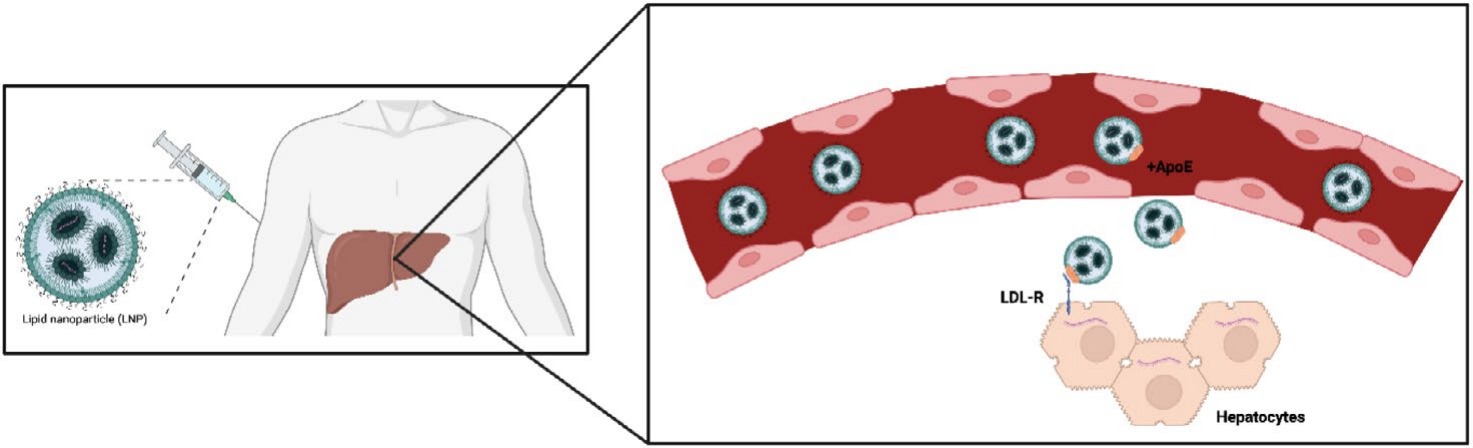
Apolipoprotein E opsonization via systemic delivery. Systemic delivery of lipid nanoparticles (LNPs) leads to accumulation in liver cells due to the fenestrated vasculature. Opsonization of the plasma protein Apolipoprotein E to LNPs and subsequent binding to low‐density lipoprotein receptor (LDLR) mediates internalization by hepatocytes.

**FIGURE 3:**
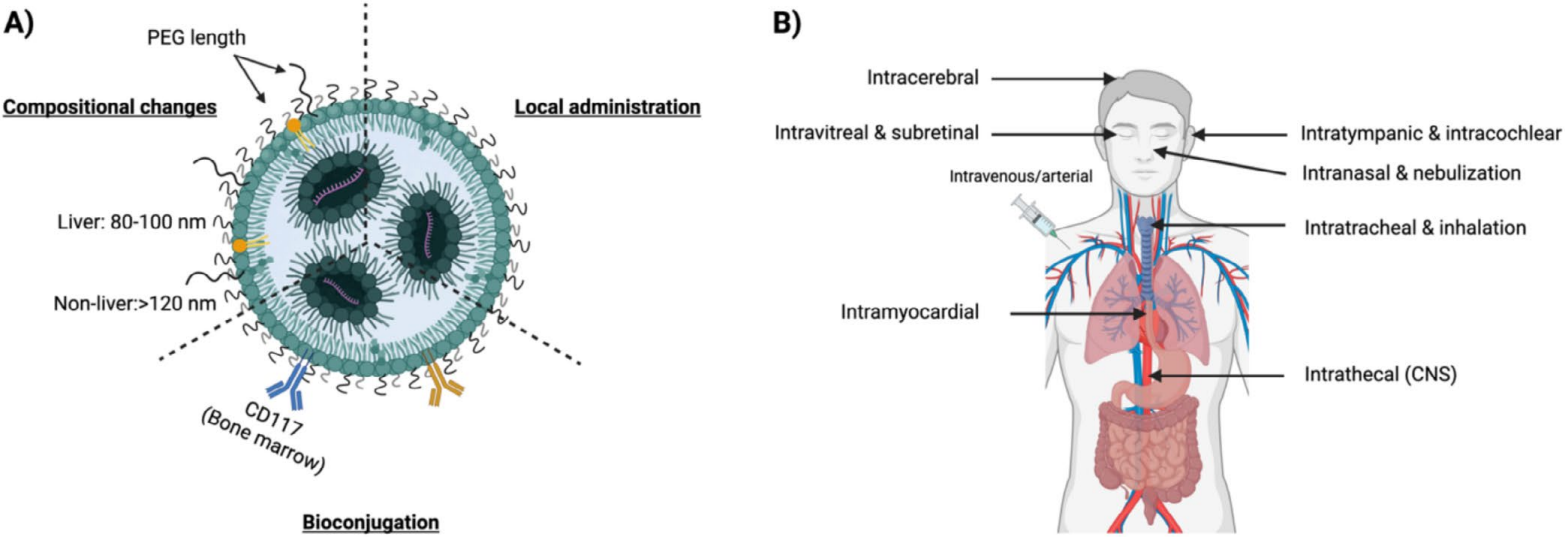
Extrahepatic delivery and local administration routes. (A) Extrahepatic delivery can be accomplished via three strategies: (i) compositional change of the lipid nanoparticle, (ii) bioconjugation of peptide to the lipid nanoparticle, and (iii) via local administration. (B) Main local administration routes for the delivery of lipid nanoparticle‐encapsulated mRNA for the treatment of rare diseases.

The capacity to protect mRNA and facilitate cellular uptake combined with the ease of production through microfluidic mixing have positioned LNPs as frontrunners for delivery of mRNA therapies. After systemic administration, LNPs predominantly accumulate in the liver thanks to the fenestrated vasculature and the presence of Kupffer cells specialized in nanoparticle uptake (Ngo et al. 2022). In addition, most LNPs bind a biomolecular corona including ApoE upon dissociation of PEG2000‐DMGs (Berger et al. 2023). ApoE‐binding to LNPs stimulates natural transport pathways of lipoproteins such as low‐density lipoprotein (LDL) to hepatic tissues and LNP uptake by hepatocytes (Figure 2) (Berger et al. 2023; Breda et al. 2023; Sebastiani et al. 2021). However, there are other mechanisms and surface properties that affect organ and cell distribution, which remain relatively unknown. Compositional changes, decoration with targeting ligands, and alternative administration routes may offer possibilities to improve organ‐specific delivery. The contribution of each of these factors to LNP tropism will be discussed in the next paragraphs (Figure 3A).

#### 2.4.1 Surface Properties

The surface properties of LNPs encompass their steric conformation, charge, and the formation of a corona. These properties are influenced by the overall LNP lipid composition. The surface charge of LNPs determines interactions with plasma proteins and directly affects biodistribution, cellular uptake, and immune recognition (Akinc et al. 2010; L. Li et al. 2022). The negatively charged cell membranes repel LNPs with negative surface charges, preventing uptake by the cells. Conversely, positively charged LNPs may disrupt cellular membranes, leading to cytotoxicity (Kedmi et al. 2010). Adjusting the lipid composition to optimize the surface charge has been employed to target uptake of LNPs to specific tissues. In a recent study, this strategy enabled a shift from liver‐directed delivery to the spleen and lung. This was achieved through incorporation of a ‘SORT’ molecule, in this case a cationic lipid. A gradual increase in the percentage of cationic lipids initially directed LNP distribution to the liver, which shifted to the spleen and lungs with higher percentages (Cheng et al. 2020; Wei et al. 2023). However, cationic induced lung tropism is also associated with negative side effects, including thrombosis in the lungs and other organs. Incorporation of anionic lipids has also been shown to increase splenic accumulation of LNPs (Cheng et al. 2020). Although the mechanisms underlying such charge‐dependent organ tropism remain elusive, it has been postulated that the cationic and anionic SORT lipids attract specific proteins to the LNP corona which improve LNP binding to cells in spleen and lungs (Dilliard et al. 2021). To allow brain targeting, 12 distinct LNPs with various ILs were screened for transfection and transport across an artificial blood–brain barrier (BBB) model (Han et al. 2024). The five most promising LNPs, those including SM‐102 and DLin‐MC3‐DMA, were subsequently evaluated in mice. Following intravenous administration, C12‐494 resulted in high luciferase signal in the brain (1% of total signal), surpassing SM‐102 and DLin‐MC3‐DMA for this specific application and demonstrating a 1000‐fold increase compared to the least effective LNP. C12‐494‐containing LNPs also demonstrated elevated signals in the heart, lungs, kidney, and spleen compared to other LNPs (Han et al. 2024). Although the relative percentages compared to liver remained low, these studies underscore the significance of LNP composition for targeted delivery (Cheng et al. 2020; Han et al. 2024). This concurs with the finding that incorporation of covalent lipid species into the base‐4‐lipid LNP formulation allowed for specific tropism for at least 14 different bone marrow cell types in healthy, sickle cell disease and acute myeloid leukemia mouse models (Lian et al. 2024).

Conjugation of targeting ligands to LNPs offers another approach to enhance extra‐hepatic targeting. Organ‐specific nanobodies or antibodies can be attached to the LNP surface, for example, by chemically coupling thiol groups in the ligand to a reactive maleimide that is conjugated to a PEG lipid. This provides an anchor for incorporation in the LNP, enabling specific recognition and binding to cell surface receptors or antigens (Dammes et al. 2021). This targeted delivery strategy enables LNPs to selectively engage with desired cell types or tissues. A recent example is the use of anti‐human CD117‐LNPs to target bone marrow cells. CD117 is found on short‐ and long‐term hematopoietic stem cells (HSCs) and on certain hematopoietic progenitors. CD117‐LNPs carrying mRNA encoding a base editor and guide RNA (gRNA) targeted to a sickle cell disease‐causing mutation achieved genomic editing in 88% of cells and a corresponding increase of the β‐like globin protein with 91.7% (Breda et al. 2023). Another study in mice and nonhuman primates focused on retinal cells using MH42‐conjugated LNPs to transfect photoreceptors (PRs), retinal pigment epithelium cells (RPEs), and Müller glia after subretinal injection (Herrera‐Barrera et al. 2023). Decoration of LNPs with targeting ligands provides a promising approach for extra‐hepatic delivery, although the liver will remain a challenging barrier to evade for most nanoparticles.

#### 2.4.2 Size and Size Distribution

Size and size distribution determine LNP degradation, clearance, internalization, and biodistribution (S. Chen et al. 2016; S.‐D. Li and Huang 2008). Typically, LNP size is expressed as the Z‐average by dynamic light scattering (DLS), with dimensions generally falling within the range of 20–200 nm (Trevaskis et al. 2015). Different applications necessitate diverse particle sizes. To target the liver, a diameter of 80–100 nm is optimal, allowing particle passage through liver fenestrae, which have an approximate diameter of 100 nm (S. Chen et al. 2016). LNPs larger than 120 nm cannot penetrate these fenestrae, promoting extra‐hepatic targeting (S. Chen et al. 2016; M. Kim et al. 2021). In general, smaller LNPs, obtained by inserting a higher percentage of PEG lipids, have less cellular interactions and remain longer in circulation (Harashima and Kiwada 1996; Yathindranath et al. 2024). However, LNPs smaller than 20–30 nm are susceptible to lipid dissociation and renal clearance, compromising their effectiveness (S. Chen et al. 2014, 2016; Y. Huang et al. 2021). Advancements in rapid mixing techniques have resulted in more homogeneous particle production. Size uniformity minimizes biological variability and facilitates more comprehensive investigations into size and associated pharmacokinetics (Evers et al. 2018; Eygeris et al. 2022).

#### 2.4.3 Extrahepatic Delivery via Local Administration

For targeting other organs than the liver, local administration can be considered through direct application or injection of LNPs into specific target tissues or anatomical sites (Figure 3B). This approach allows for higher local concentrations of LNPs and more efficient delivery to nearby cells or tissues of interest. By bypassing or delaying systemic circulation, local administration reduces potential off‐target effects, offering a promising option for delivery of LNPs to extra‐hepatic tissues. Simultaneously, local administration offers a way to evade physical barriers like the blood–brain barrier.

An example of local administration includes intramyocardially administered mRNA‐LNPs in myocardial‐infarcted mice leading to transfection of cardiac cells including fibroblasts, endothelial, and epicardial cells (Labonia et al. 2024). Various studies have reported successful transfection of the retina after local administration in the eye of LNP‐delivered mRNA. In mice, subretinal injection of LNP‐mRNA led to successful transfection (16%–27%) of PRs and the RPE (Gautam et al. 2023), which was confirmed in both mice and nonhuman primates (Herrera‐Barrera et al. 2023).

However, local delivery is not applicable for every organ. Often, only the cells surrounding the injection site are reached upon local administration, which may make local therapy relatively ineffective or not feasible. For example, many (> 10) intramyocardial injections of naked mRNA were required in the infarct area of human patients. For diseases such as Duchenne Muscular Dystrophy, where multiple muscles in different anatomical sites are affected, local administration may only be beneficial to (part of) a single muscle and require a combination of local and systemic delivery. There is also a risk of unintended targeting of surrounding tissue with less precision. Because local administration and the resulting relatively high local concentrations can also magnify the toxicity of nucleic acid therapies (Labonia et al. 2024), dosing and treatment should be optimized for each organ and application.

Another strategy for extrahepatic delivery via local administration is ex vivo gene editing followed by autologous transplantation. This approach involves isolating specific cells from the patient, editing them outside the body, and then transplanting them back. It is already clinically feasible for editing diseased HSCs from the bone marrow and has potential for other organs as well (Sheridan 2023).

Although significant strides have been made to achieve extrahepatic delivery of mRNA‐LNPs, it is important to realize that hepatic delivery still dominates.

### 2.5 Virus‐Like Particles for Therapeutic mRNA Delivery

The limitations of LNPs to reach organs beyond the liver have catalyzed the exploration of other delivery platforms. Recently, VLPs have emerged as an interesting alternative. VLPs are nanoscale virus‐like structures with the coat but without the genetic core of viruses, thus retaining the effective viral cell‐targeting and transduction properties without the risks of genotoxic effects. Until recently, it was difficult to engineer VLPs with sufficiently efficient cargo loading, but with the new generation VLPs, cargo loading and delivery efficiency have increased to putative therapeutic levels (M. An et al. 2024; Banskota et al. 2022; Hamilton et al. 2021; Mangeot et al. 2019; Segel et al. 2021).

#### 2.5.1 Structural Properties—Cargo Loading

**FIGURE 4:**
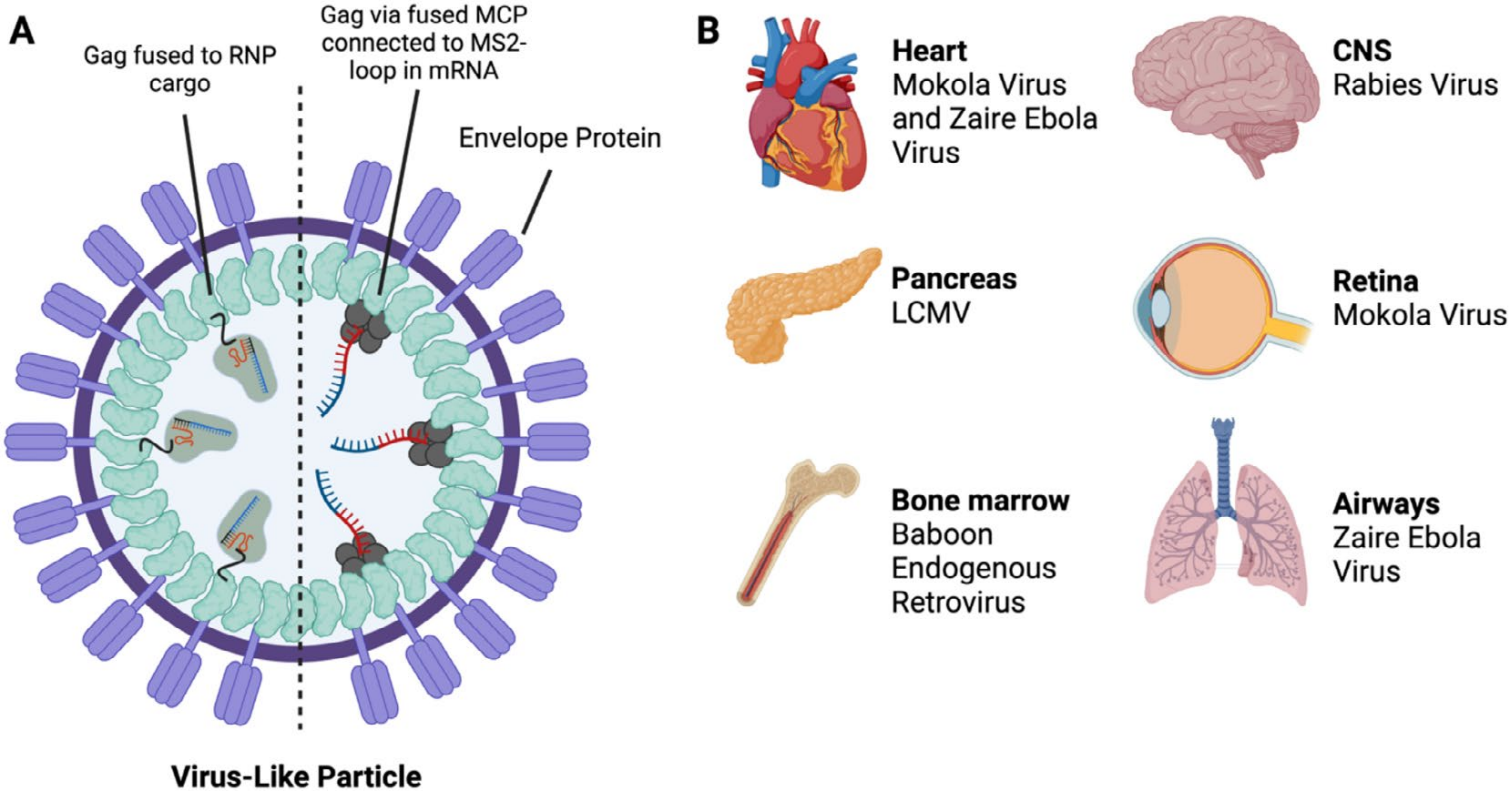
Viral tropism and pseudotyping of VLPs for targeted organ delivery. (A) A schematic depiction of a VLP, where capsid proteins form the outer layer and Gag polyproteins recruit the cargo, which can be mRNA, protein, or RNP complex. (B) Examples of tropism of various viruses, of which coat proteins could be used to pseudotype VLPs to improve organ specificity. LCMV = lymphocytic choriomeningitis virus.

VLPs are typically produced in producer cells through the self‐assembly of viral structural proteins from both eukaryotic and prokaryotic origins. Central to this process are the Murine Leukemia Virus–derived and HIV‐derived Gag polyproteins, known for their ability to self‐assemble into VLPs (Mangeot et al. 2019; Yandrapalli et al. 2016). Together with Gag polyproteins, additional capsid proteins, such as Vesicular Stomatitis Virus G protein (VSV‐G), are incorporated to form the VLP's envelope, which enhances the capacity to fuse with cellular membranes of a wide range of target cells in a pH‐dependent fashion. Encapsulation of cargo within the VLP occurs when producer cells express cargo linked to Gag. For the delivery of proteins, the protein of interest is directly fused to Gag and self‐packaged into the VLP. For mRNA delivery, an additional step is necessary to bind the mRNA. This can be achieved by integrating a MS2 aptamer into untranslated regions of the mRNA, which can bind to the MS2 Coat Protein (MCP) that is fused to Gag, enabling loading of the mRNA into the VLP (M. An et al. 2024; Banskota et al. 2022) as visualized in (Figure 4A).

#### 2.5.2 Surface Properties—Active Targeting

In lentiviruses, the VSV‐G capsid protein facilitates the entry of viruses into cells by binding to the LDLR on the cell membrane. VSV‐G has broad tropism because the LDLR is ubiquitously expressed in most cell types (Hastie et al. 2013). VSV‐G can be substituted with capsid proteins from other viruses. This technique, called pseudotyping, is used in virology to change the tropism of a virus, thereby enabling it to enter cell types it would not naturally infect.

Pseudotyping with the rabies virus glycoprotein (RVG) led to efficient delivery of RNP constructs to brain cells in mice after cerebroventricular injection (Banskota et al. 2022). By extrapolation, the surface proteins of specific organ‐targeting viruses can be used to design VLPs that can target essentially any organ of interest. Examples include the Baboon endogenous retroviral envelope glycoprotein to target HSCs (Morizono et al. 2005), Zaire Ebola pseudotypes to target lung epithelial cells and cardiomyocytes (Fe Medina 2003), lymphocytic choriomeningitis virus (LCMV) glycoproteins for insulin‐secreting beta cells from the pancreas (Kobinger et al. 2004), and Mokola viral glycoproteins for retinal cells and cardiomyocytes (Auricchio et al. 2001; Bemelmans et al. 2005; MacKenzie et al. 2002), as depicted in Figure 4B.

To further improve cell‐specific targeting, integration strategies are developed to immobilize targeting antibodies or genetically engineered antibody mimicking proteins (DARPINS) on the VLP envelope. This has been shown to be compatible with VLPs, leading to effective targeting of CD117, which is highly expressed on the surface of HSCs and induces endocytosis (Strebinger et al. 2023). With the current speed of developments in this field, other promising approaches will undoubtedly emerge soon.

The viral coat proteins of VLPs can induce immune responses. Although this may hinder efficient delivery and function of the cargo, it could be beneficial for application as vaccines. A famous example is Cervarix, a VLP‐based vaccine against HPV16 and HPV18. This VLP formulation contains the HPV capsid L1 protein, which elicits potent antiviral immunity (Szarewski 2010). Undesired immunogenicity of capsid proteins on VLPs may limit their use for the treatment of genetic diseases to a single administration to avoid excessive immune responses (Fuchs et al. 2015; Nooraei et al. 2021). In AAV clinical trials, pre‐exposure to the virus is an exclusion criterion to mitigate immune responses that could compromise therapeutic efficacy, as mentioned in NCT02122952 (Al‐Zaidy and Mendell 2019; Colella et al. 2018; Naveed and Calderon 2021). Nonetheless, preclinical data associated with a recently initiated clinical trial demonstrated that repeated administration of the lentiviral‐based gene therapy was well tolerated without triggering a host immune response (NCT06515002) (Allen et al. 2023). While reducing the immunogenicity of viral proteins is becoming an increasingly active field of research by developing humanized variants (Segel et al. 2021), the immunological consequences of repeated administration of the current generation of VLPs still remain to be evaluated.

Another factor to consider is the production method of VLPs using producer cells in batches, given the unfeasibility of long‐term expression of some VLP components (e.g., fusogens). Upon overexpression of luciferase in producer cells, luciferase was also included in VLPs and in targeted cells (Mangeot et al. 2019), and the same may hold true for other cytoplasmic content. However, similar batch production methods are used for viral gene therapies, and this has not posed significant problems. For clinical use, VLP purity and batch reproducibility will still be important factors to consider.

### 2.6 Alternative Delivery Methods

LNPs have emerged as leading candidates in clinical mRNA delivery due to their efficient delivery and favorable safety profile. However, challenges such as inflammatory components and their predominant tropism for the liver limit their broader applicability. VLPs offer a bio‐inspired alternative with potential advantages, particularly their improved delivery efficiency and cell–type specificity. Nonetheless, they also have immunogenicity challenges and primarily accumulate in the liver upon systemic administration. Subtle differences in cell‐type specificity may become apparent when comparing VLPs to LNPs, particularly under local administration (Banskota et al. 2022).

In contrast, polymer‐based mRNA delivery systems, although under development for a long time, have not yet achieved widespread clinical use like LNPs. This is primarily due to their toxicity and low transfection efficiency (Fischer et al. 2003; W. Yang et al. 2023). Despite these challenges, polymer‐based systems exhibit unique features. These include their capacity to form a variety of nanostructures in aqueous environments and distinct pharmacokinetic profiles (e.g., extended circulation time) that could advance mRNA therapeutics (Guerrero‐Cázares et al. 2014). Poly(lactic‐co‐glycolic acid) (PLGA), for example, is an FDA‐approved polyester utilized in drug delivery (W. Yang et al. 2023).

Another noteworthy polymeric platform includes charge‐altering releasable transporters (CARTs), a polymeric‐lipid hybrid system (Z. Li et al. 2023; McKinlay et al. 2017). These block oligomers consist of an initiator, one or more lipid blocks, and a polycationic block. The cationic component of CARTs forms electrostatic complexes with polyanions under acidic conditions (pH < 5.5). At physiological pH, an acyl shift converts the polycation to a neutral lactam, facilitating the release of the mRNA cargo—an essential step for ensuring the mRNA is free for efficient translation while minimizing cytoplasmic toxicity caused by high cationic charges (Z. Li et al. 2023; McKinlay et al. 2017). However, under static buffer conditions at pH 7.4 the acyl shift progresses slowly due to the absence of cellular cues required to promote full dissociation of the mRNA‐polymer complex. Consequently, CARTs remain largely intact in this environment. In living cells, the dynamic biochemical environment plays a crucial role: the endosomal acidification followed by the return to near‐neutral pH in the cytoplasm creates the conditions necessary to trigger the acyl shift. This process transforms cationic amines into neutral amides (or lactams), reducing the polymer's positive charge and promoting the release of mRNA for translation (Z. Li et al. 2023; McKinlay et al. 2017).

Optimizing CART structures have resulted in transfection rates of up to 70% in primary T cells in vitro. Additionally, systemic in vivo delivery in mice demonstrated up to 97% tropism for the spleen and transfected 8% of splenic T cells without requiring T‐cell–specific targeting ligands (Z. Li et al. 2023; McKinlay et al. 2017). Although CARTs have a significantly improved transfection profile, they still face considerable challenges, including off‐target interactions, immunogenicity, stability concerns, and difficulties controlling release (Fischer et al. 2003; Z. Li et al. 2023; McKinlay et al. 2017).

Extracellular vesicles (EVs) also present a compelling alternative delivery method due to their biocompatibility, low immunogenicity, and natural ability to mediate cellular communication. However, their practical use is limited by significant challenges, including upscaling, difficulty in loading cargo, heterogeneity, and a lack of understanding of their delivery mechanisms. Addressing these hurdles is crucial for unlocking their full therapeutic potential (Bader et al. 2024).

## 3 Design of mRNA for Therapeutic Application

### 3.1 Structural Aspects to Enhance mRNA Translation and Function

Once the delivery vehicle has successfully transported mRNA into the target cells, the mRNA faces new intracellular challenges. Upon cellular uptake into endosomes, mRNA must escape from the endosomes to avoid sequestration and degradation by lysosomal enzymes. After endosomal release into the cytoplasm, mRNA contends with stability and immunogenicity challenges presented by the intracellular environment. To improve stability and reduce immunogenicity for therapeutic use, mRNA is generally produced using in vitro transcription (IVF) with several general mRNA modifications to produce modified mRNA (modRNA).

#### 3.1.1 The Untranslated Regions (UTRs)

**FIGURE 5:**
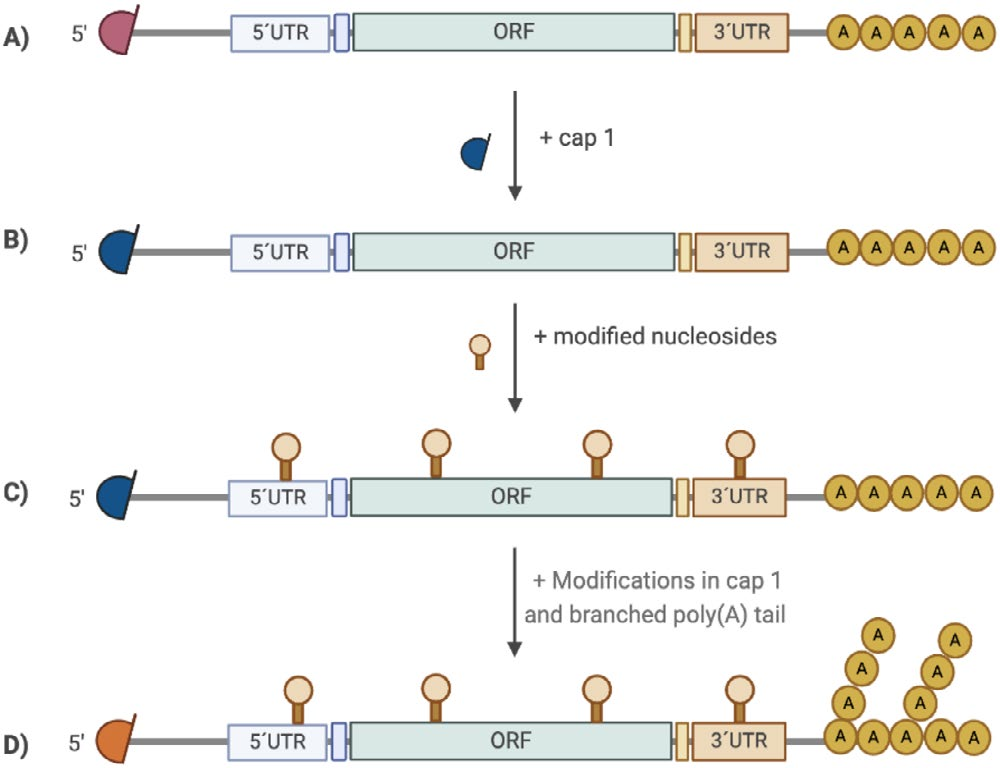
Schematic representation of key structural elements of IVT‐derived mRNA. (A) Unmodified mRNA with Cap 0. (B) Introduction of Cap 1 analogs into mRNA structure to increase stability and translation efficiency. (C) Substitution of nucleosides with chemically modified nucleosides to decrease immunogenicity and increase stability and translation efficiency. (D) Chemical modifications in the Cap 1 analog and introduction of branched poly(A) structure to increase yield and protein expression.

Within the structure of mRNA (Figure 5A) the 5′ and 3′ UTRs regulate translation (initiation, elongation and termination) and stability (Asrani et al. 2018; S. C. Kim et al. 2022; Suknuntha et al. 2018; Zarghampoor et al. 2019). Within the 5′ UTR, start codons and noncanonical start codons hinder transcription of the desired mRNA, therefore they should be removed from therapeutic mRNAs. The secondary structure of the 5′ UTR can also impact translation of the resulting mRNA. Highly stable secondary structures prevent ribosome recruitment and codon recognition (Asrani et al. 2018; Leppek et al. 2018; Sonenberg and Hinnebusch 2009). As an example, RNA G‐quadruplexes and hairpins directly hinder ribosome loading and scanning (Jia et al. 2020), while stem‐loop structures enhance translation (Jia et al. 2020; Jia and Qian 2021).

Similarly, the 3′ UTR design is important for mRNA translatability. The fundamental strategy is to use sequences from endogenous mRNAs associated with high protein translation and extended half‐life, such as those found in α‐globin and albumin (Asrani et al. 2018; Zarghampoor et al. 2019). Most eukaryotic mRNAs contain mRNA degradation signals in the 3′ UTR to regulate mRNA stability. For instance, AU‐rich sequences in the 3′ UTR participate in the removal of the poly(A) tail, leading to degradation of the mRNA. To enhance stability, substitution of these AU‐rich sequences with sequences from more stable mRNAs has been proposed (Asrani et al. 2018; Barreau 2005; Eberhardt et al. 2007).

#### 3.1.2 The 5′ Cap

Before translation can start, a pre‐initiation complex is formed between the 5′ cap and the eukaryotic translation initiation factor 4E (EIF4E). Subsequently, the small (40S) ribosomal subunit loaded with methionyl tRNA specialized for initiation interacts with this complex and starts scanning the 5′ UTR to reach the start codon (Jia and Qian 2021; Sahin et al. 2014; Sonenberg and Hinnebusch 2009; To and Cho 2021; Zarghampoor et al. 2019). Natural eukaryotic mRNA has a 7‐methylguanosine (m7G) cap which interacts with EIF4E. However, IVT mRNA results in an uncapped 5′ triphosphate which is vulnerable to exonuclease‐mediated degradation, which compromises bioavailability and is associated with immune reactions (Oh and Kessler 2018; Sahin et al. 2014). For therapeutic use, a suitable 5′ cap is thus required. The two main methods to produce the desired 5′ cap in IVT mRNA involve enzymatic capping, requiring a secondary enzymatic treatment following the IVT reaction, or co‐transcriptional capping, incorporating a synthetic cap analog during the IVT reaction (Henderson et al. 2021; Sahin et al. 2014).

Enzymatic capping is attractive due to the high capping efficiency compared to co‐transcriptional capping with an anti‐reverse cap analog (ARCA). The ARCA competes with GTP for incorporation, resulting in approximately 30% remaining uncapped mRNA (Vaidyanathan et al. 2018). However co‐transcriptional capping reduces manufacturing steps, subsequently lowering costs and simplifying mRNA production. Different generations of cap analogs have been developed. The first generations resulted in Cap 0 with low yields and low capping efficiency (Henderson et al. 2021; Vaidyanathan et al. 2018). Both uncapped and Cap 0 mRNA can stimulate immune responses through recognition by the innate immune receptor RIG‐1 (Jia and Qian 2021; To and Cho 2021). The next generation (Cap 1) involved a methyl group introduced to the ribose 2′‐O position of the first transcribed nucleotide. This Cap 1 structure (Mauer et al. 2017) prevents degradation of mRNA transcripts and plays an important role in the distinction between self and non‐self‐RNAs (Figure 5B) (Jia and Qian 2021; Picard‐Jean et al. 2018; To and Cho 2021; Vaidyanathan et al. 2018). Different Cap 1 analogs have now been introduced for co‐transcriptional capping. An example is CleanCap AG trimer with higher efficiency and higher yields of capped mRNA (Henderson et al. 2021) due to additional modifications to the basic structure of the cap, including a 3′ O‐methylation on m7G and more recently also methylation in Position 6 of the first adenosine. Thanks to increased resistance to mRNA‐decapping enzymes, these modifications increase protein translation and RNA stability (Mauer et al. 2017).

#### 3.1.3 The Open Reading Frame (ORF)

The ORF corresponds to the part of the mRNA encoding the desired protein. Codon optimization can improve translation of the encoded mRNA. Replacement of rare codons by common codons enhances translation efficiency. However, this must be carefully assessed on a case‐by‐case basis, as certain proteins require slow translation to achieve proper folding and stability, which is facilitated by rare codons. Accordingly, some mRNA‐encoded vaccines retain the original ORF for optimal performance (Angov et al. 2008; Sahin et al. 2014; To and Cho 2021). An algorithm called “codon harmonization” was developed to identify sequences that require slower translation (Angov et al. 2008), which may help to select optimal codons for a specific mRNA.

Exposure to non‐self‐nucleic acids triggers the mammalian innate immune system via Toll‐like receptor activation. In the context of vaccination, this intrinsic immunostimulatory effect and adjuvant activity of unmodified IVT mRNA may be beneficial (Karikó et al. 2008; Oh and Kessler 2018; Sahin et al. 2014), but for most other applications, immunogenicity is a disadvantage. To address this, Karikó and Weissmann introduced modified nucleosides (Figure 5C) (Karikó et al. 2005, 2008). Most commonly employed modified nucleosides are *N*6‐methyladenosine (m6A), inosine, *N*1‐methyladenosine (m1A), pseudouridine (Ψ), methylpseudouridine, 5‐methyl cytidine (5meC), and 5‐hydroxymethylcytosine (5hmC) (Vaidyanathan et al. 2018). Complete replacement of uridine for pseudouridine was found to increase protein expression 10‐fold in cell lines and primary cells (Karikó et al. 2008), which was attributed to reduced activation of protein kinase R (Anderson et al. 2010), reduced Toll‐like receptor signaling (Karikó et al. 2005) and decreased activation of 2′–5′‐oligoadenylate synthetases (Anderson et al. 2011). *N*1‐methylpseudouridine substitution provided even better translation and in vivo performance, making it currently the most commonly used modification in IVT mRNA (Andries et al. 2015).

Despite the evident benefits in terms of immunogenicity and protein translation, modified nucleosides may interfere with ribosomal recognition of the correct reading frame during translation. Recently it was described that incorporation of N1‐methylpseudouridine in IVT mRNA induces a +1 ribosomal frameshift both in vitro and in vivo. It was hypothesized that for the COVID vaccines, this mistranslation could lead to the presentation of +1 frameshifted antigens to T cells, potentially triggering off‐target cellular immune responses. Although no adverse reports have been associated with this mistranslation in mRNA‐based COVID vaccines, it underscores the importance of sequence optimization to prevent potential off‐target effects for future mRNA‐based therapeutics (Mulroney et al. 2024).

#### 3.1.4 The 3′ Poly(A) Tail

The 3′ poly(A) tail has an important role in translation initiation and mRNA stability (Baptista et al. 2021; Dreyfus and Régnier 2002). Most actively translated mRNAs in mammalian cells have a poly (A) tail containing 100–250 adenosine residues (Oh and Kessler 2018; Peng et al. 2008; To and Cho 2021). This poly (A) tail binds to polyadenosyl‐binding proteins (PABP), which subsequently interact with the N‐terminal region of EIF4G. This complex interacts with the 5′ cap and EIF4E complex, forming a circularized mRNA structure. This circularization protects the mRNA from nucleolytic degradation and enhances translation efficiency by ribosome reutilization (H. Chen et al. 2024; Oh and Kessler 2018; Peng et al. 2008; To and Cho 2021; Zarghampoor et al. 2019). In general, translation efficiency is proportional to the number of adenosines in a single tail (Oh and Kessler 2018; Peng et al. 2008; To and Cho 2021). Recent findings suggest that the multimerization of the 3′ poly (A) tail via a branched topology (Figure 5D) protects it against RNA decay and preserves interactions with PABP for prolonged translation. Chemical modification of these multimers further improved protein expression and function at minimal doses in both cell lines and mice (H. Chen et al. 2024).

## 4 Therapeutic Application of mRNA

**FIGURE 6:**
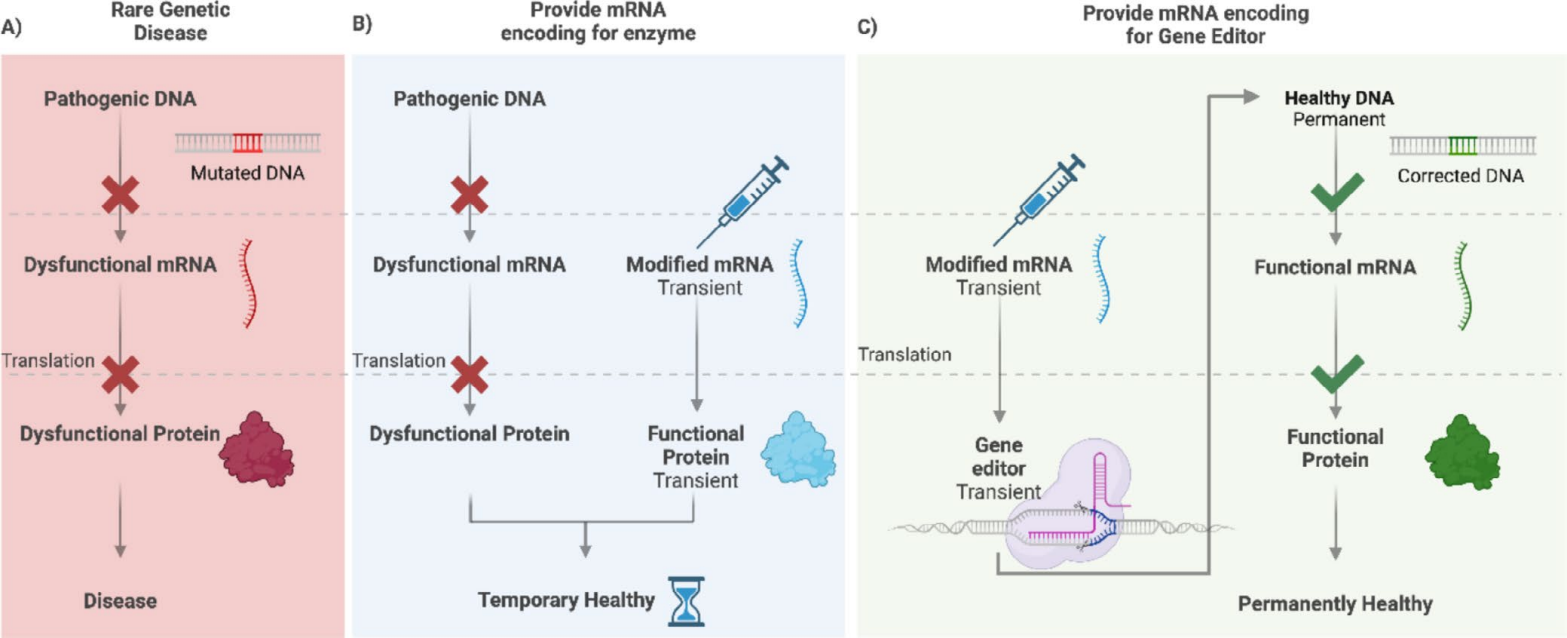
Therapeutic strategies for rare genetic diseases using modified mRNA. (A) Pathogenesis of a rare genetic disease: mutated DNA leads to the production of dysfunctional or deficient mRNA and consequently a dysfunctional or deficient protein, resulting in disease. (B) Therapeutic application of mRNA: Modified mRNA encoding for a functional enzyme is introduced into the cell. Upon translation, this mRNA facilitates the production of the functional protein, which can restore healthy cellular function. (C) Modified mRNAs encoding for the gene editor and the guide RNA are delivered to the cell. This gene editor corrects the mutated DNA, resulting in permanently corrected DNA, enabling the cell to produce functional mRNA and subsequently a functional protein, thus addressing the origin of the disease.

mRNA is an interesting molecule for rare diseases caused by genetic mutations that lead to the production of defective or deficient proteins (Figure 6A). mRNA therapies can act as substitutes for these defective or deficient proteins by supplying cells with healthy mRNA encoding these proteins (Figure 6B). However, due to the transient lifespan of both the mRNA template and the resulting protein, these treatments provide only transient expression of the enzyme that is chronically missing or deficient (Pardi et al. 2015). The transient expression of mRNA can also be harnessed for a permanent solution by providing CRISPR gene‐editing tools as mRNA in combination with appropriate gRNAs as the “come‐and‐go tools” to repair disease‐causing DNA mutations (Figure 6C).

### 4.1 mRNA to Restitute a Deficient Enzyme

Feasibility of mRNA substitution therapy depends on the type of disease (continuous versus acute episodic enzyme deficiency), the half‐life of the mRNA and encoded protein, the organs and cell types in which the enzyme should be expressed, and the accessibility of these organs. For some diseases, targeting a single organ or cell type can revert the disease phenotype, as illustrated by diseases that can be treated with an organ transplantation, such as liver transplantation for, for example, Wilson's disease, glycogen storage disease, and Crigler–Najjar syndrome and HSC transplantation for, for example, sickle cell disease and severe combined immunodeficiency (Finotti et al. 2021; Khan et al. 2021). These organs may now also serve as targets for mRNA therapies (D'Antiga et al. 2023; Gilgenkrantz 1995; Schene et al. 2020). Since delivery of functional mRNA to the target organ is currently the main determinant for the efficiency of mRNA therapies, clinical therapies are guided by the accessibility of organ targeting with currently available delivery vehicles and administration routes (Fumoto et al. 2013).

As shown in Table 1, the current clinical pipeline for systemic delivery of mRNA therapies focuses on the liver. Targeted diseases are metabolic diseases such as organic acidemia, glycogen storage disease, urea cycle defects, and phenylketonuria, and involve biweekly or triweekly intravenous administration (D. An et al. 2017, 2019). Based on preclinical research, the bone marrow (Shi et al. 2023) and lungs (Qiu et al. 2022) may also be targeted via intravenous administration of LNPs. Inhalation of mRNA in LNPs is now studied to target the lungs in cystic fibrosis.

**TABLE 1:** Current clinical trial pipeline for mRNA substitution therapies.

Drug name	Disease	Target organ	Delivery tool	Trial number
mRNA‐3927	Propionic acidemia	Liver	mRNA‐LNP	NCT04159103
mRNA‐3704	Methylmalonic acidemia	Liver	mRNA‐LNP	NCT03810690
mRNA‐3745	Glycogen storage disease 1a	Liver	mRNA‐LNP	NCT05095727
mRNA‐3210	Phenylketonuria	Liver	mRNA‐LNP	NCT06147856
MRT5201	Ornithine transcarbamylase deficiency	Liver	mRNA‐LNP	NCT03767270
MRT5005	Cystic fibrosis	Respiratory tract	mRNA‐LNP	NCT03375047
mRNA‐3692/VX‐522	Cystic fibrosis	Respiratory tract	mRNA‐LNP	NCT05668741

### 4.2 mRNA Encoding for Gene‐Editing Tools

To exploit transient expression of mRNA for a permanent solution, CRISPR gene‐editing tools may be administered as mRNA in combination with appropriate (pe)gRNAs to repair disease‐causing DNA mutations (Figure 6C). By repairing the root cause of monogenic diseases, this would revolutionize prospects for patients with genetic diseases. Current modalities for CRISPR‐Cas9 delivery involve plasmid DNA, mRNA, or RNP complexes (Kouranova et al. 2016). Each of these modalities has its own advantages and disadvantages. The transient expression of mRNA is attractive for gene‐correction therapies because it enables establishment of a permanent gene edit, after which the mRNAs and guide RNAs are naturally degraded and eliminated from the cell (Glass et al. 2018), thereby preventing persistent activity of the Cas9 nuclease and limiting unwanted editing effects (S. Kim et al. 2014; Ramakrishna et al. 2014).

The first generation of gene‐editing tools typically comprised a Cas9 protein and a gRNA (Jinek et al. 2012). The Cas9 protein creates a double‐stranded break (DSB) at a genomic target location determined by the gRNA. The DSB triggers the cell's DNA damage response mechanisms. These mechanisms can be exploited to introduce a desired DNA sequence (homology directed repair), but more often result in nonhomologous end joining (NHEJ) of the DNA breaks. NHEJ is not very precise and often leads to nucleotide insertions and deletions, which generally inactivate genes. This strategy of NHEJ is exploited in the first approved treatments for β‐thalassemia and sickle cell disease (Casgevy). These two diseases are caused by mutations in the hemoglobin subunit B (HBB) gene, thereby disrupting the production of functional hemoglobin (Harteveld et al. 2022). Instead of repairing these mutations, Casgevy is a Cas9/sgRNA RNP complex that is introduced ex vivo into hematopoietic stem or progenitor cells by electroporation to deactivate BCL11A, a gene responsible for repressing fetal hemoglobin production after birth. Suppression of fetal hemoglobin is required after birth to transition to adult hemoglobin for improved oxygen transport. However, in individuals with β‐thalassemia or sickle cell disease, fetal hemoglobin can compensate for the deficient adult hemoglobin. Clinically, Casgevy indeed inactivated BCL11A, re‐enabling production of fetal hemoglobin and alleviating the symptoms without immunological or adverse events (Sheridan 2023). In addition to Casgevy, other mRNA‐based CRISPR/Cas9 therapeutics are now registered for clinical trials, as described in Table 2.

**TABLE 2:** Current clinical (trial) pipeline for gene‐editing therapies.

Drug name	Disease	Target organ	Gene editor	Cargo	Delivery tool	Trial number
**Ex vivo**						
Casgevy	Sickle cell disease B‐thalassemia	Hematopoietic stem cells	Cas9	RNP	Electroporation	NCT05477563
EDIT‐301	Sickle cell disease B‐thalassemia	Hematopoietic stem cells	Cas12a	RNP	Electroporation	NCT04853576
Beam‐101	Sickle cell disease, B‐thalassemia	Hematopoietic stem cells	Base editing	Unknown	Electroporation	NCT05456880
CTX211	Type 1 diabetes mellitus	Pancreas	Cas9	Unknown	Unknown	NCT05565248
PM359	Chronic granulomatous disease	HSC	Prime editing	Unknown	Unknown	NCT06559176
**In vivo**						
NTLA‐2001	Transthyretin (ATTR) Amyloidosis	Liver	Cas9	mRNA	LNP	NCT06128629
NTLA‐2002	Hereditary Angioedema	Liver	Cas9	mRNA	LNP	NCT05120830
VERVE‐101 VERVE‐102	Heterozygous familial hypercholesterolemia	Liver	Base editing	mRNA	LNP	NCT05565248 NCT06164730
VERVE‐201	Refractory Hyperlipidemia	Liver	Base editing	mRNA	LNP	NCT06451770
Beam‐301	Glycogen storage disease 1a	Liver	Base editing	mRNA	LNP	NCT06735755
Beam‐302	Alpha‐1 antitrypsin deficiency	Liver	Base editing	mRNA	LNP	NCT06389877
CTX310	Atherosclerotic cardiovascular disease	Liver	Cas9	mRNA	LNP	NCT identifier pending
CTX320	Lp(a) for cardiovascular disease.	Liver	Cas9	mRNA	LNP	NCT identifier pending
ECUR‐506	Ornithine transcarbamylase deficiency	Liver	ARCUS nuclease	DNA	AAV	NCT06255782
SB‐318	MPS‐1	Liver	ZFN nuclease	DNA	AAV	NCT02702115
EBT‐101	HIV‐1 Infection	CD4+ T cells	Cas9	DNA	AAV	NCT05144386, NCT05143307
EDIT‐101	Leber Amaurosis	Retina	Cas9	DNA	AAV	NCT03872479
ZVS203e	Retinitis Pigmentosa	Retina	Cas9	DNA	AAV	NCT05805007
BD111	Viral Keratitis	Retina	Cas9	mRNA	LV^1^	NCT04560790

Abbreviation: LV, lentiviral particle.

While conventional CRISPR/Cas9 is effective in knocking out genes, it is less efficient in correcting disease‐causing mutations using HDR. Given the fact that 90% of rare genetic diseases are caused by single base substitutions (Lavrov et al. 2020), next‐generation gene editors were developed to enable precise corrections at the single‐base level (Komor et al. 2016). In 2015, David Liu's group introduced base editors to deaminate and convert specific nucleotides into the desired nucleotides. By engineering the cutting Cas9 into a nicking Cas9, single‐strand DNA breaks are introduced, which do not activate the DNA repair mechanisms associated with DSB that cause unwanted insertions and deletions. Currently, base editors can address 6 of the 12 possible base substitutions, theoretically enabling correction of approximately 73% of all pathogenic single‐nucleotide polymorphisms (SNPs) as cataloged in the ClinVar database (Gaudelli et al. 2017; Rees and Liu 2018). However, the other base substitutions and the remaining types of mutations, such as deletions and insertions, remain unaddressed by base editing. Also of concern is the unwanted conversion of non‐targeted bases in the base editor's 6‐nucleotide editing window (Sánchez‐Rivera et al. 2022), and the introduction of gRNA‐independent off‐target effects such as genotoxic translocations (Fiumara et al. 2023; M. E. Huang et al. 2024). Nevertheless, when off‐target editing is mitigated by the development of more efficient protospacers, base editing holds significant clinical promise. This is exemplified by the first‐in‐human base editing trial targeting the PCSK9 gene in the liver with VERVE‐101 mRNA‐LNPs (NCT05565248). The encoded base editor converts an adenine into a guanine nucleotide to inactivate the PCSK9 gene, resulting in lowered plasma cholesterol levels. The trial's clinical outcomes indeed demonstrate a 73% reduction in low‐density lipoprotein cholesterol (LDL‐C), underscoring the potential of in vivo base editing (Robinson and Kosikowski 2023). However, safety concerns emerged after one participant experienced a myocardial infarction potentially related to the treatment and another participant with severe underlying heart disease died from a heart attack, which was deemed unrelated to the treatment. After another Grade 3 adverse event, Verve Therapeutics decided to prioritize VERVE‐102, which uses the same base‐editing strategy with another LNP involving an ionizable lipid with a GalNAc liver‐targeting ligand (NCT06164730).

Prime editing stands out as a cutting‐edge advancement in gene editing, offering the precision and versatility that addresses several limitations of base editing (Anzalone et al. 2019; P. J. Chen and Liu 2023; Doman et al. 2022). This method employs a Cas9 nickase fused to a reverse transcriptase, guided to the target DNA location by a prime editing guide RNA (pegRNA). Unlike base editing, prime editing does not present the same on‐target (no bystander editing) and off‐target safety risks (Tao et al. 2023). Prime editing theoretically holds the capacity to correct all types of smaller genetic variations. However, the technique is still in the early stages of development, and it remains challenging to devise efficient prime‐editing tools for each different mutation, often requiring screening many pegRNA/prime editor combinations. Furthermore, CRISPR tools and particularly prime editors are large and do not fit in viral vectors, making mRNA delivery via LNPs and VLPs particularly desirable. This field is advancing rapidly with the ongoing evolution of newer, more efficient, and smaller prime editors (Doman et al. 2023; Lu et al. 2022; Yan et al. 2024). Various animal studies have already demonstrated that prime editing can correct both genotype and phenotype in vivo with a single treatment. Given the potential for a one‐time application, more invasive administration routes become a feasible option to reach difficult‐to‐target organs. This is exemplified by local injections into the retina or brain in animal models, showcasing the method's promise for treating a broad spectrum of genetic conditions with precision and safety (M. An et al. 2024; Davis et al. 2024).

First‐generation gene editors are now tested in clinical trials. A wave of base‐editing trials is already underway, and the first prime‐editing trials recently entered Phase 1 (Table 2). Advances in delivery tools will broaden the scope of target organs beyond the liver and HSCs, thereby increasing the range of rare genetic diseases eligible for treatment.

Industrial development of gene‐editing therapies for (ultra) rare diseases presents significant challenges due to high investment costs and limited revenue potential from a narrow patient population. It is not surprising that current gene‐editing approaches predominantly focus on gene inactivation rather than gene correction. This strategy can be used to treat a diverging number of diseases (like sickle cell disease and thalassemia) caused by different individual mutations and thereby expand the market. Gene correction poses a more complex scenario, requiring tailored therapies for each individual disease and each individual mutation, which is particularly impractical for diseases caused by many different mutations such as cystic fibrosis, for which more than 2000 different mutations have been identified (Veit et al. 2016). Each individual mutation would require its own (pe)gRNA and lengthy optimization trajectory. In this respect, mRNA therapies for enzyme replacement (Table 1) may be attractive because they restore enzyme activity irrespective of the disease‐causing mutation. However, most genetic diseases are chronic, and efficient treatment requires continuous enzyme activity. The commercial benefits of a chronic repetitive off‐the‐shelf patients treatment should be carefully balanced versus the personal and societal advantages of a permanent solution provided by a single therapy, which requires extensive individualized development. By sharing the responsibility between regulators, industry leaders, and scientists to advance these therapies as a platform technology, developmental time and costs can be reduced. Additionally, providing a regulatory framework that allows for accelerated approval for treating different mutations in the same genes, in analogy to the rapid development of new mRNA vaccines for evolving COVID‐19 variants, may lead to the development of accessible gene‐editing therapies for patients with rare genetic diseases.

## 5 Conclusion and Future Perspectives

For decades, the use of mRNA as a therapeutic agent remained challenging. Recent developments have now positioned mRNA therapies as extremely promising strategies for rare genetic diseases. The main challenges associated with mRNA stability and immunogenicity have been tackled, although delivery challenges persist. A significant breakthrough was established by the development of LNPs for functional delivery of mRNA, enabling the development of the highly successful mRNA vaccines. In the extracellular space, LNPs protect mRNA from degradation and immune recognition. Once inside the cell, immunogenicity is further prevented by mRNA modifications. These same modifications enhance stability and translation, leading to the first clinical successes of mRNA therapeutics. For mRNA‐based enzyme replacement, these advances in mRNA modifications can extend the expression duration of mRNA. This will reduce administration frequency in future mRNA treatments, which will positively impact immunogenicity and patient burden. The current mRNA therapeutic pipeline is dictated by the feasibility to reach specific organs and cell types. For rare genetic diseases, intravenous mRNA‐LNPs are mainly used to efficiently target the liver. Local administration and decoration of LNPs with cell‐specific nanobodies will also allow targeting of other organs (e.g., the eye after subretinal injection), the lung (after inhalation) and specific cell types (e.g., HSCs). Targeting other organs is still an ongoing challenge. In addition to LNPs, VLPs are being developed to target specific organs. Capsid proteins of VLPs can be pseudotyped to mimic the evolutionary capability of viruses to infect specific organs and cell types, as exemplified by the RVG pseudotype to target brain cells. Although pseudotyping is an established concept, there is still limited published research on pseudotyped VLPs for organ‐specific cargo delivery (as opposed to vaccination).

Together, these developments have led to the first therapeutic applications of mRNA‐based enzyme replacement and gene‐editing therapies for rare genetic diseases. While enzyme replacement therapy provides only a temporary solution for patients with rare genetic diseases, gene editing offers the promise of a permanent cure. The first generation of gene editors was suited for gene inactivation rather than gene correction. To get there, a gene‐editing technique that can efficiently correct every individual mutation is necessary. Prime editing emerges as the most suitable candidate for this purpose, although improvements in versatility and efficiency are essential to accelerate and maximize clinical translation. The current speed of development of new prime editors is fast, with smaller, more efficient, and more versatile prime editors arising every year. It is tempting to consider the possibilities once organ‐specific targeting with mRNA is achievable. Upon solving the challenge of “getting there,” mRNA therapies may well revolutionize prospects for patients with rare genetic diseases.

## Author Contributions

**Paul J. L. Schürmann:** conceptualization (lead), methodology (lead), visualization (lead), writing – original draft (lead), writing – review and editing (equal). **Stijn P. E. van Breda Vriesman:** conceptualization (supporting), writing – original draft (supporting), writing – review and editing (supporting). **Jose A. Castro‐Alpízar:** conceptualization (supporting), writing – original draft (supporting), writing – review and editing (supporting). **Sander A. A. Kooijmans:** writing – original draft (supporting), writing – review and editing (supporting). **Edward E. S. Nieuwenhuis:** conceptualization (equal), writing – original draft (supporting), writing – review and editing (lead). **Raymond M. Schiffelers:** conceptualization (lead), funding acquisition (lead), writing – original draft (equal), writing – review and editing (lead). **Sabine A. Fuchs:** conceptualization (lead), writing – original draft (equal), writing – review and editing (lead).

## Conflicts of Interest

R.M.S. is currently vice president of preclinical R&D of Nanocell Therapeutics.

## Related WIREs Articles

An overview of lipid constituents in lipid nanoparticle mRNA delivery systems

## Data Availability

Data sharing is not applicable.
